# Skills Developed by Student Pharmacists Completing a Research Project in the Doctor of Pharmacy Program

**DOI:** 10.3390/pharmacy14040097

**Published:** 2026-07-02

**Authors:** David R. Axon, Becka Eckert, Alyx Meilinger, Maren Steffen, Haylee Bingham, Justin Pacheco, Houston Swann, Kayleen Tubbs

**Affiliations:** 1 James L. Winkle College of Pharmacy, University of Cincinnati, Cincinnati, OH 45267, USA; 2 R. Ken Coit College of Pharmacy, University of Arizona, Tucson, AZ 85718, USA; beckert@arizona.edu (B.E.); alyxmeilinger@arizona.edu (A.M.); marenksteffen@arizona.edu (M.S.); hayleebingham@arizona.edu (H.B.); justinpacheco@arizona.edu (J.P.); houstonlswann@arizona.edu (H.S.); kayleentubbs@arizona.edu (K.T.)

**Keywords:** pharmacy research, research project, student research skills, curricular revision

## Abstract

Objective: To explore fourth-year student pharmacists’ perspectives of the skills they develop by completing a research project in the pharmacy curriculum at one United States College of Pharmacy. Methods: Semi-structured interviews were conducted with a sample of fourth-year student pharmacists until thematic saturation was achieved. Interviews were recorded and transcribed verbatim. Thematic analysis was conducted to identify main themes. All records were reviewed by two researchers, and consensus was sought at each step of the analysis. Results: Eleven interviews were conducted. Three themes related to skills developed by students while completing a research project were apparent from the final reconciled code list: Theme 1—critical analysis skills; Theme 2—residency preparedness skills; and Theme 3—interpersonal skills. Critical analysis skills helped students review the literature and appropriately translate the findings into clinical settings. Residency preparedness skills described how students perceived completing a research project would prepare them for a residency program. Interpersonal skills were inter-related and consisted of collaboration, communication, conflict resolution, leadership, and project management skills. Conclusions: These study findings should be considered by curriculum committees as changes to the pharmacy program are considered. Future research is suggested to explore other perspectives of the research process.

## 1. Introduction

At the University of Arizona R. Ken Coit College of Pharmacy, the curriculum committee has recurringly discussed whether a research project should continue to be mandatory for all student pharmacists. The Accreditation Council for Pharmacy Education (ACPE) Standards 2025 dictate the need for student pharmacists to learn about statistics and study design, but do not explicitly state that student pharmacists must perform their own research project [1]. Pharmacy curricula are typically very content-heavy with requests for ever more topics to be included. As such, it is necessary to have conversations about how much content, and what content, can, and should be taught in United States (US) Doctor of Pharmacy (PharmD) programs. Several recent papers have been published discussing various aspects of curricular hoarding, curricular overload, and efforts for curriculum simplification, among others [2,3,4,5,6,7]. For instance, evidence from a recent review suggests that a more efficient, well-planned curriculum, or a curriculum with reduced workload for student pharmacists, may offer opportunities for better understanding of taught content and improved well-being and satisfaction [8]. For student pharmacists who seek to pursue further training with a research component, such as residency, fellowship, or even graduate studies, there may be considerable value in conducting a research project as part of their PharmD education. For students planning to pursue a pharmacy career elsewhere, a research project may be less beneficial, at least in the context of limited time in the PharmD curriculum. These student pharmacists may prefer to prioritize their time on something more suited to their career goals.

Recent reports have described various aspects of research projects in the PharmD curriculum in the US. For instance, one report described an elective project opportunity that involved six credit hours of work across the second and third years of the PharmD program, culminating in a written project proposal, oral proposal presentation, written report, and poster presentation [9]. Another report outlined how student pharmacists could pursue an elective research project without credit throughout their fourth year, which also involved a poster presentation [10]. A further report described how student pharmacists in the third and fourth years of the PharmD program were required to complete a required research project, with a required poster and research report as the course deliverables [11].

Although there is some literature describing research projects in US pharmacy curricula as summarized by a recent review [12], there is no contemporary documented literature examining student pharmacists’ experiences of completing a research project. In particular, there is a lack of literature exploring the skills student pharmacists say they develop as a result of completing a research project. The purpose of this paper is to report upon fourth-year student pharmacists’ perspectives of the skills they develop by completing a research project in the pharmacy curriculum at the University of Arizona R. Ken Coit College of Pharmacy. The information gleaned from this study may be helpful to inform revisions to the pharmacy curriculum at the University of Arizona R. Ken Coit College of Pharmacy or to others who are considering adding, removing, or revising a pharmacy research project component in the curriculum at their institution.

## 2. Materials and Methods

This study involved qualitative semi-structured interviews with fourth-year student pharmacists currently undertaking their research project at the University of Arizona R. Ken Coit College of Pharmacy. The research project is a required part of the PharmD curriculum at this institution. Students identify a project and project advisor and can work independently or select their own teams of up to five students. The research project course series runs across the final three semesters of the four-year PharmD program. In the Spring semester of the third year, student pharmacists take an in-person course that teaches them how to write a proposal for their research project and prepare to submit an Institutional Review Board (IRB) application. This course is taught by the pharmacy project coordinator (a PhD-trained pharmacist faculty member) predominantly from the primary (Tucson) campus, with a live-feed to student pharmacists at the satellite (Phoenix) campus. Throughout the semester, the instructor visits the satellite (Phoenix) campus to meet and support those student pharmacists, with additional online meetings as necessary. Throughout the fourth year, alongside their Advanced Pharmacy Practice Experiences (APPEs), student pharmacists obtain the necessary project approvals and then carry out their research project under the supervision of their project advisor. The pharmacy project coordinator provides support as needed. In the final week of the fourth-year Spring semester, student pharmacists present a poster of their findings to their peers.

The research team developed a semi-structured interview guide specific for this project that contained a brief two-minute overview of the project, including the purpose of conducting the project, and how the interviews would work. The interview guide consisted of five core questions with additional probing questions that could be used as necessary related to the aims of the study (Table 1), including a final opportunity to mention anything else about research in the pharmacy program that was not already covered during the interview. The interview guide contained a prompt for the interviewer to invite any questions about the interview process and to seek informed consent before the interviews begun. Finally, the interview guide contained a seven-item demographic questionnaire to be completed at the end of the interview (age, sex, campus, education before pharmacy school, grade point average [GPA] during the pharmacy program, previous experience with research, and whether the student pharmacist worked independently or in a group for their project). The semi-structured interview guide was developed and iteratively refined through group discussion based on the existing published literature and several years’ experience teaching student pharmacists to complete research projects [13,14,15,16].

All 126 fourth-year student pharmacists (split approximately evenly between the primary (Tucson) campus and the satellite (Phoenix) campus) were invited to participate in this study via e-mail, with additional reminder e-mails sent to recruit student pharmacists until data saturation was met. Most student pharmacists, unless they completed their project early, would have been conducting their projects at the time of the interviews. All student pharmacists who agreed to participate were interviewed until saturation was met. Interviews were conducted from Winter 2024 through Spring 2025 with the graduating class cohort of 2025. No incentives (financial, grades, or otherwise) were offered to participate in this study. Student pharmacists were reminded that in all aspects of the study, their participation was voluntary, and their responses would be anonymous. Interviews were led by a neutral researcher (BE) who was not involved in the student pharmacists’ education with the intent that they would feel comfortable providing honest perspectives. Student pharmacist researchers also assisted with the interviews (HB, HS, JP, KT). These student pharmacists were working ahead of schedule and used this project to meet the research project requirement of the PharmD curriculum but were not in the same cohort as the student pharmacists eligible for inclusion in this study. These researchers had been appropriately trained in conducting qualitative semi-structured interviews. Interviews were conducted online via zoom at a mutually convenient day and time, were audio-recorded, and later transcribed verbatim for analysis. Thematic analysis was conducted by two researchers (AM, MS) working independently, with input from additional researchers (BE, DRA) as necessary. The thematic analysis utilized the Braun and Clarke approach [17,18]. This involved the researchers reading the transcripts independently to familiarize themselves with the content and creating two code lists in Dedoose (Version 10.0.35, Los Angeles, CA, USA: SocioCultural Research Consultants, LLC.) [19], that were later reconciled through discussion until consensus of a final code list was met. Core themes were identified from the final codes, which were reviewed to explore patterns and connections between them and to check that the themes were appropriately interpreted. The themes were named and described alongside representative excerpts from the interview transcriptions in the report [17,18].

## 3. Results

### 3.1. Demographic Characteristics of Study Participants

Eleven student pharmacists shared their perspectives of research projects in the PharmD program through semi-structured interviews. See Table 2 for the demographic characteristics of the interviewees. Interviews ranged from 10 to 29 min, with an average length of 21 min (±6.8 min).

### 3.2. Themes Identified in Thematic Analysis

Three themes related to skills developed by student pharmacists while completing a research project were apparent from the final reconciled code list: Theme 1—Critical Analysis Skills; Theme 2—Residency Preparedness Skills; and Theme 3—Interpersonal Skills. These themes are described below with representative excerpts from the interviews.

### 3.3. Theme 1: Critical Analysis Skills

The first theme focuses on the critical analysis skills developed by student pharmacists as they completed a research project in the PharmD program. In particular, how these skills were honed by reviewing the extant research literature, distinguishing the quality and appropriateness of the study design and its findings, and determining how to appropriately translate the findings into the clinical setting. For instance, one interviewee described how they developed their critical analysis skills as they explored the literature: “I think it [the research project] prepares us because pharmacy is constantly evolving, and especially with the amount of literature that’s being produced… I know how to critically analyze data for myself, because I had to do that for my project” (Interview 8).

Building on this, a second interviewee described how their critical analysis skills helped them accurately use clinical guidelines, as well as knowing when to use (or not use) information found in a study: “Doing a research project exposes us to reading research, and seeing how those guidelines are formed, and it kind of gives us a better understanding of how to use those guidelines in practice, and also being able to differentiate between a good research study versus bad. I think that’s super beneficial” (Interview 4).

A third interviewee highlighted how their critical analysis skills were important to help understand new information produced from research and translate it into patient-centered care: “I definitely just want to take everything that I’ve learned and, you know, how can we make things better, with this new information out… how are we going to provide this and kind of simplify it for our patient” (Interview 2)?

### 3.4. Theme 2: Residency Preparedness Skills

The second theme focused on the student pharmacists’ residency preparedness skills. Interviewees discussed how completing a research project helped prepare them for a future residency program. One interviewee illustrated how completing a research project would make them more competitive in the residency application process: “I think that experience [completing a research project] was just something that every pharmacy student needs to go through… it was a great thing to put on our CV. Especially for those of us applying to residency. It definitely put us a step ahead of some other applicants from different schools who may not have had as much research experience as we did” (Interview 6).

A second interviewee described how their research project gave them something to discuss during their residency interviews: “[This research project is] something that I know that I am able to showcase during these interviews, and I’d be proud to talk about how my team and I were able to complete this research project in our fourth year” (Interview 9).

Another interviewee focused on how the skills gained during their research project would be applicable for their residency program: “In residency, you’re expected to evaluate a lot of literature, so having that background of how a study is formed, developed, how data is analyzed, I think, is very important as most residency programs require you to complete a research project… so, having that experience and not having it be my first time during residency, will definitely prove helpful. Even though it will be a different project, I kind of understand what the process is now” (Interview 11).

### 3.5. Theme 3: Interpersonal Skills

The final theme focused on their interpersonal skills. Several interpersonal skills were identified, including collaboration, communication, conflict resolution, leadership, and project management skills. The representative quotes provided by participants for this theme (see Table 3) indicated that these interpersonal skills often overlapped with each other.

Interviewees mentioned how completing a research project encouraged them to collaborate as they worked towards a shared goal by leveraging the different thought processes and skill sets of team members. In one case, the interviewee described how collaboration skills gained from completing a research project may help with (inter-) professional collaboration in the future. Another interviewee reflected that their research project collaboration helped them gain experience managing the various personalities of team members.

Interviewees noted that working on a group research project provided them with an opportunity to develop and refine their communication skills. This included their scientific writing skills, as well as their oral presentation skills. Of note, interviewees described how completing a research project afforded them an opportunity to develop their public speaking skills by presenting their project at national conferences, such as the American Society of Health-System Pharmacists (ASHP) Midyear Clinical Meeting. In one case, an interviewee explained how they learned to adapt their communication style for different scenarios throughout the course of the project. Often related to collaboration and communication, interviewees described instances where they developed their conflict resolution skills, typically through “tough conversations”.

One interviewee described gaining leadership skills through proactive communication and organization, while another recounted how their leadership skills evolved to meet the requirements of a particular situation. Finally, interviewees described how the research project helped them improve their project management skills. One interviewee discussed how careful delegation and distribution of tasks facilitated each group member to trust each other in creating quality work. Similarly, another interviewee outlined how managing tasks and the time needed to put into them was important in the overall course of the project. Of note, interviewees stated the importance of project and time management in order to complete their research project in the available time. This involved finding time to work on the project alongside their other commitments such as Advanced Pharmacy Practice Experience (APPE) rotations or work commitments.

## 4. Discussion

This qualitative study identified the skills developed by student pharmacists while completing a research project as part of the PharmD curriculum. Several skills were identified in the interviews that warrant discussion. These skills should be considered by the curriculum committee at the R. Ken Coit College of Pharmacy, as changes to the PharmD program are considered. These findings may also be of interest to other institutions who are preparing to add, remove, or change a pharmacy research project component in the PharmD curriculum when considering the skills that students may obtain from a similar educational experience.

First, it was encouraging that student pharmacists identified the value of research in the PharmD curriculum. In particular, student pharmacists noted how completing a research project helped develop their drug literature evaluation skills, enabling them to better understand how to identify relevant research reports in the literature, and appropriately critique their quality. Student pharmacists also mentioned how identifying and evaluating this information was important for creating clinical guidelines and providing evidence-based, patient-centered care. This is important because pharmacists practicing in a variety of settings often have to answer questions regarding new medications and supplements as well as read studies to help inform their therapeutic recommendations. Completion of a research project may help improve student pharmacists’ abilities to interpret, implement, and communicate clinical research into practice, contributing to high-quality patient care. These critical analysis and clinical reasoning skills gained have been corroborated in previous research as a major benefit of participation in research [20].

Second, many student pharmacists spoke about the value of completing a research project for residency preparedness. Since most residency programs require residents to complete a research project during their training, student pharmacists in these interviews expressed how the execution of a research project during pharmacy school equips them with knowledge and familiarity with the research process. In addition, student pharmacists in this study perceived that completion of a research project made them more competitive for residency positions. Previous work has noted research as a key element of the residency preparation checklist and has demonstrated that student pharmacists who have graduated from public institutions with a focus on research were more likely to match with residency programs than student pharmacists from other institutions [21]. However, the perceived importance of research skills is not consistent across residency directors and may be dependent on the area of practice [22].

Third, student pharmacists identified a range of interpersonal skills that they obtained by completing a research project in the PharmD program. Examples of these skills included collaboration, communication, conflict resolution, leadership, and project management. Some of these skills have been outlined in the ACPE Standards 2025 as core competencies for student pharmacists to develop throughout the PharmD curriculum [1]. It is likely that these skills would be more strongly identified among student pharmacists working as part of a group to complete their project, although student pharmacists working independently without other student pharmacists may still have developed these skills through interactions with their project advisor and any other project collaborators.

Effective collaboration is essential in pharmacy practice, as pharmacists regularly coordinate with physicians, nurses, and other healthcare providers to ensure optimal patient care [23]. Student pharmacists are expected to practice this skill of engaging and contributing as healthcare team members to deliver high-quality care [1]. Completing a research project is an involved process that requires several team members performing specific roles, and collaboration is necessary to effectively manage each component of the research project. In this study, participants described meaningful experiences in working with peers, faculty mentors, and interdisciplinary partners. By participating in a research team, student pharmacists engaged in the iterative processes of delegation and collective problem-solving, directly paralleling real-world pharmacy team dynamics.

Communication is a critical aspect of patient-centered medicine and is advanced through conversations encountered in activities such as group research projects. Learning how to sufficiently communicate with peers in a research setting can provide the foundation for contributing to an interdisciplinary team in other settings. Student pharmacists appreciated the opportunity to present the findings of their projects at a national meeting, which can help develop their communication and networking skills. Many student pharmacists plan to attend professional meetings and being able to present their research project adds additional value to their participation at such events. Encouragingly, student pharmacists shared their pride and satisfaction at being able to share their research results with the pharmacy community, including their student peers, faculty, and new connections. Aside from these benefits, attending professional meetings affords student pharmacists nearing the end of their studies an opportunity to explore new areas of pharmacy practice and potential next steps in their careers.

Alongside collaboration and communication, student pharmacists noted how completing the research project helped them develop their conflict resolution skills. This may be a valuable skill for student pharmacists to develop and apply in their future clinical practice. The ability to resolve conflicts professionally is critical in high-stakes clinical environments where disagreements about medication therapy or care plans may arise [24].

Furthermore, participants described taking on leadership roles within their research teams, stepping into positions that required them to guide peers, initiate solutions, and represent the group in communications with faculty. As the role of the pharmacist continues to evolve, effective leadership is essential to promote behavioral and systems change, while advocating for both patients and the profession. Research has identified a persistent gap in leadership qualities within pharmacy practice and urges development of these skills to inspire future pharmacy leaders [25].

Managing the many components of a research project offered student pharmacists an opportunity to develop project management skills. Goal setting, time management, and accountability tracking are all important skills for practicing pharmacists, especially for those who may pursue clinical trial research positions [26]. Student pharmacists who mastered project timelines and adapted to unforeseen challenges during their research projects may be better equipped to handle workflow interruptions or emergent priorities in clinical settings.

Although these are important skills for pharmacists to obtain, there are likely other areas of the PharmD curriculum (and other academic, extra-curricular, life, and work experiences) where student pharmacists can develop and refine these skills, such as interprofessional and team-based learning experiences [27,28,29]. However, execution of an ongoing longitudinal project over several semesters does afford student pharmacists an opportunity to work on refining these skills over time. Future research is needed to explore if there are any differences in the development of these skills between student pharmacists who completed a research project in the PharmD curriculum and those who did not.

As with all studies, this qualitative analysis of interviews has some limitations. Although this study had a reasonable sample size for qualitative research, it was not intended to produce generalizable results. Thus, the results may not apply to other colleges or schools of pharmacy. A brief description of the research project course set up at this institution is provided in the methods to assist readers in determining if this scenario is similar or not to their home institution. The study is cross-sectional in nature, reflecting the views of students who undertook the same series of research courses at one college of pharmacy. Given that the interviews were conducted while the student pharmacists were undertaking their projects, it is possible that the findings may be different if the interviews were conducted after the projects were completed, or after some time has passed (e.g., after completing a residency program or the next stage of their careers). Further research may be warranted to explore the findings in future. The interpretation of this qualitative analysis may be influenced by the researchers’ existing understanding or perceptions of the topic, but the independent coding and team consensus discussions should help mitigate this possible bias.

While this interview study provides some interesting insights into the skills developed while conducting a research project in the PharmD curriculum from the student perspective, the findings also highlight avenues for further exploration. Future research could be conducted exploring some of the challenges students experienced, as well as student pharmacists’ perceptions of their research project advisor. Research may also be conducted on research projects in the curriculum from the faculty perspective. Additional research may be warranted to quantify the improvement of skills and compare research-driven skill development to that of other types of experiences. Finally, the impact of any changes made to the PharmD curriculum with regard to research projects should be evaluated in the future. Given the lack of any other published studies exploring student pharmacists’ perspectives of the skills gained when completing a research project in the pharmacy program, the findings of the current study may offer comparison data for the results of future studies.

## 5. Conclusions

This qualitative semi-structured interview study identified three key themes from student pharmacists about the skills they developed while completing a research project in the PharmD curriculum. These included critical analysis skills, residency preparedness skills, and interpersonal skills. These findings should be considered by curriculum committees, at the college where this study was conducted, or other institutions who are considering adding, removing, or revising a pharmacy research project component in the curriculum, as changes to the pharmacy program are considered. Future research is suggested to explore other perspectives of the research process.

## Figures and Tables

**Table 1:** Core questions asked in the semi-structed interview guide with example probing questions.

Core Questions	Example Probing Questions
To begin with, please tell me a bit about your experience completing a research project in the pharmacy program.	N/A
There are many tasks that are required when completing a research project, from forming a team and coming up with a research question, developing a proposal, obtaining IRB approval, collecting and analyzing data, and disseminating the results. Could you tell me how you went about completing these tasks?	Any particular strategies that were used?
How did completing a research project contribute to your professional development?	Can you discuss any specific skills or competencies you feel you have developed or strengthened through this research project?
How important or valuable is it for pharmacy students to complete a research project?	How do you think completing a research project enhances the overall educational experience for pharmacy students?
Is there anything else you’d like to tell me that you think is important about research projects in the pharmacy curriculum that I haven’t asked about?	N/A

**Table 2:** Demographic characteristics of study participants.

Interviewee	Age	Gender	Campus Location	Education Background	GPA During Pharmacy Program	Have You Worked on Any Other Research Project (Other than the Quality Improvement Project) Before Your Doctor of Pharmacy Research Project? *	Did You Work Independently or as Part of a Group for Your Doctor of Pharmacy Research Project?
1	25–29	Female	Phoenix	Associate’s + Bachelor’s	3.25–3.49	No	Group
2	25–29	Male	Phoenix	Associate’s + Bachelor’s	3.50–3.74	No	Group
3	25–29	Female	Phoenix	Bachelor’s	3.25–3.49	No	Group
4	25–29	Male	Phoenix	Bachelor’s	3.25–3.49	No	Group
5	≥30	Female	Phoenix	Associate’s	I wish not to answer	No	Group
6	20–24	Female	Tucson	N/A	3.75–3.99	No	Group
7	I wish not to answer	Female	Phoenix	Associate’s	3.75–3.99	No	Group
8	25–29	Female	Tucson	Bachelor’s	4.0	No	Group
9	25–29	Female	Phoenix	Bachelor’s	3.75–3.99	Yes	Group
10	≥30	Female	Phoenix	Associate’s	3.25–3.49	No	Group
11	20–24	Female	Tucson	N/A	3.75–3.99	Yes	Group

* All students previously completed a quality improvement project as part of the pharmacy curriculum.

**Table 3:** Quotes representative of Theme 3: Interpersonal Skills.

Interpersonal Skills	Quote
Collaboration	“It gives you more experience working in a group… because as pharmacists we will have to work with other pharmacists or other healthcare professionals.” (Interview 6)
Collaboration/Conflict resolution	“I think there are benefits [of working in a group]… navigating different personalities helps prepare us for future situations.” (Interview 3)
Communication	It [the research project] also helps with writing skills and everything that you essentially will need to be successful as a pharmacist.” (Interview 8)
Communication	“The other part is the public speaking portion of it, just because me and one of my group members did present at Midyear… so I think the public speaking portion of it really helped, and being able to talk to pharmacists and researchers about our project was super validating and rewarding. I think it has helped me to be able to do that in the future again.” (Interview 4)
Communication/Conflict resolution	“I kind of had to learn how to change how I communicate, and I feel like that’s going to help me in the future, like being able to know how to communicate better with different circumstances.” (Interview 1)
Conflict resolution/Communication	“Individuals are fearful of having those professional conversations, but sometimes that’s actually needed… and it refocuses the group. I think [it’s important to] be courageous enough to express how you feel and have tough conversations… they’re really beneficial and eliminates any assumptions made by team members and any type of animosity that could build up for lack of communication.” (Interview 2)
Leadership/Communication	“I think leadership was also a skill I picked up. I was kind of in charge of communication for my team– reminding people of deadlines, setting up our Google drive where all the information was going to be. I learned a lot of leadership and communication from that.” (Interview 9)
Leadership/Conflict resolution	“Leadership as well played a big role, because sometimes some people would say, ‘Oh, like, I don’t want to take this part. I don’t want to do that.’ So that pushed me to be a leader in specific areas of the project, like, it’s okay that some people don’t want to work on that. I will take the lead.” (Interview 7)
Project management/Communication	“[Our team] assigned a chunk to each person, and you just have to make sure you get your chunk done. You trust your teammates are going to get it done… and we all read through each other’s parts to make sure it looks good and give constructive feedback.” (Interview 6)
Project management	“You really have to find a way to do your project while also working 40 h at rotation and just find time to analyze data, whether that be on the weekends or after rotation. And then, especially if you’re working too… you just have to manage that. So, I would say it does help you grow professionally as well.” (Interview 8)

## Data Availability

The raw data supporting the conclusions of this article will be made available by the authors on request.
